# The Importance of an Emotional Expression Guide to Prevent Work-Related Health Problems in Emotional Laborers

**DOI:** 10.3390/ijerph18136710

**Published:** 2021-06-22

**Authors:** Ji Sun Ha, Jin Ah Kim

**Affiliations:** 1 College of Nursing Science, Kyung Hee University, 26 Kyungheedae-ro, Dongdaemun-gu, Seoul 02447, Korea; hajs@khu.ac.kr; 2 Department of Nursing, Honam University, 120 Honamdae-gil, Gwangsan-gu, Gwangju 62399, Korea

**Keywords:** emotional labor, emotional expression guide, occupational health

## Abstract

Background: As the service industry develops, the proportion of emotional laborers is gradually increasing, and their occupational health problems are gradually becoming serious social problems. Researchers must consider various factors, from the personal to the organizational levels, to prevent health problems from arising in the workplace. Many intervention studies have investigated the health and wellbeing of workers, but mainly at the individual level, even though an organization’s interest and efforts are essential for addressing work-related health problems. Therefore, the purpose of this study was to verify the importance of organizations’ interests to protect emotional laborers from work-related health problems. Methods: We used data obtained through the 4th Korean Working Condition Survey of 2014. The study cohort comprised 5857 survey participants over the age of 18 years. Employers, self-employed persons and professional soldiers were excluded. Logistic regression was employed to identify associations between an emotional expression guide and work-related health problems using SPSS 22.0 statistical software. Results: In the absence of an emotional expression guide, the risk of work-related physical and psychological health problems was increased. Even after adjusting for confounding variables, the risks were statistically maintained, particularly headache (odds ratio (OR) 1.798; 95% confidence interval 95% CI: 1.288–2.508), lower limb muscular pain (OR: 1.627; 95% CI: 1.130–2.342), general fatigue (OR: 1.582; 95% CI: 1.077–2.326) and depressive symptom (OR: 6.149; 95% CI: 1.198–31.563). Conclusion: This study showed that organizations’ interests and efforts to prevent workers from being harmed by the effects of emotional labor are important in the prevention of psychosocial and physical health problems; therefore, a national interest in supporting emotional laborers and in introducing policies to support these workers should be established.

## 1. Introduction

Emotional labor refers to carrying out work in a manner desired by the employer, regardless of the employee’s feelings [1]. With the expansion of the service industry, companies have actively sought to improve customer service to better compete with others, placing further emotional labor on their employees. In a report on the status of emotional workers in Korea [2], emotional workers were defined as persons who deal directly with customers for more than half of their working hours. This report showed that the number of emotional workers in Korea is estimated to be 43.5% of all (17 million) wage workers, and that the number of workers engaged in emotional labor increases every year [2]. Emotional labor elicits emotional dissonance, as the individual must express emotions that may differ from their actual emotions, and this is known to have negative consequences such as a diminished job ability and satisfaction and emotional exhaustion [3,4]. Moreover, high emotional demands in the workplace have been shown to have negative effects on physical and psychosocial health [5]. For this reason, emotional labor is becoming an important social issue globally. Accordingly, national efforts to prevent and manage emotional labor have been attempted.

Regarding the health problems of emotional laborers, previous studies reported that, although they differ depending on the work environment, emotional laborers are more likely to have psychosocial health problems [6]. In particular, burnout is the main cause of psychosocial health problems in emotional laborers [6]. Burnout is defined as a physical, emotional and mental state observed in people, especially in those who are in constant face-to-face professional interaction with other people, involving physical fatigue, long-term exhaustion, desperation and hopelessness, as well as the concept of a negative self, feelings of inefficiency, and a negative attitude toward others [6]. Furthermore, inadequate or disrupted sleep is a common symptom among emotional laborers, which is a risk factor for a range of psychiatric disorders including anxiety and mood disorders [7,8]. Individuals who do not sleep well tend to have impaired work productivity and to consume more medical resources [9], and employees suffering from insomnia have been shown to have significantly higher rates of absenteeism and to cause increased costs for both employers and the community [10,11].

Emotional laborers’ health problems not only affect psychosocial health but also physical health, with symptoms such as backache and muscular pain [12]. Occupationally related musculoskeletal disorders are the most common occupational diseases among emotional laborers in Korea [12]. Moreover, several studies have drawn links between the risk factors of emotional labor and work-related physical health problems [12,13,14]. Physical and psychosocial health problems that emotional laborers may suffer can lead to industrial accidents. To address these issues, the Emotional Workers Protection Act was passed and went into effect in October 2018 in Korea to support emotional laborers and to implement the Emotional Workers Protection Act, a guide for emotional expression is essential for customer service workers [15]. However, emotional workers primarily work in small-sized workplaces, and the employer is often inclined to pursue company profits over worker health and safety [16]. Even if an emotional expression guide for customer service workers is not prepared, there are no legal penalties, so many workplaces have not introduced such guides [15].

According to McLeroy [17], researchers must consider various factors, from the personal to organizational levels, when conducting intervention studies to protect workers from health problems. Moreover, a previous intervention study for reducing cardiovascular disease risk factors among blue collar workers asserted that organizational interesting for worker’s health is important to decrease work-related physical and psychological health problems of workers [18]. Many intervention studies for health promotion among workers have been performed, although they have primarily been conducted at the individual level even though the organization’s interest and efforts are essential for addressing work-related health problems [19]. Therefore, an approach in the context of the organizational level, such as an emotional expression guide, is essential to prevent the negative factors that may occur due to emotional labor.

In this study, we attempted to investigate the effect of interest at the organizational level on the prevention of emotional laborers’ health problems.

## 2. Theories

### 2.1. Importance of Organizational Support: Ecological Model

Ecological models of health behavior emphasize the environmental and policy contexts of behavior while incorporating social and psychological influences. Ecological models lead to the explicit consideration of multiple levels of influence, thereby guiding the development of more comprehensive interventions [17,20]. According to McLeroy [17], researchers must consider various factors, from the personal to the organizational level in order to protect the workers from health problems. In particular, McLeroy emphasized the need to focus on policy and environmental changes to help people choose healthy behaviors [17] because providing individuals with motivation and skills to change behavior cannot be effective if environments and policies make it difficult or impossible to choose healthful behaviors [20]. However, many intervention studies that investigate health promotion among workers are primarily conducted at the individual level [19]. Therefore, researchers and health medical members should create environments and policies that are convenient and attractive, and then motivate and educate workers to make healthy choices.

Based on the ecological model, we assumed that organizational attention and support are important to protect the work-related physical and psychosocial health of emotional laborers.

### 2.2. Perceived Organizational Support

Previous studies on the effect of organizational support showed that organizational members who receive and perceive a wide variety of organizational support increased their job satisfaction and organizational commitment to the organization, and decreased turnover intention [21]. Consequently, organizational performance increased. Kim et al. stated that emotional workers who feel that they are supported by organizations can depend on organizations or supervisors when times are difficult at work [21]. Thanks to organizational support, employees may feel that the work stressor in question is less problematic, which may mitigate the contribution of the stressor to employee strain [21]. These positive effects have a great effect on improving the physical and mental health of workers. Indeed, many studies have reported that perceived organizational support can reduce the health problems of emotional workers [22,23,24,25]. Therefore, based on many previous studies related to perceived organizational support, we hypothesized that an emotional expression guide could be produced for reducing of emotional workers’ mental and physical health problems.

Based on the ecological model and perceived organizational support, in this study, we attempted to evaluate the positive effects of an emotional expression guide, which provides organizational support for emotional workers and on the health of emotional workers. Based on the results of this study, we are planning to establish prevention and intervention strategies, including organizational supports for work-related psychosocial and physical health problems caused by emotional labor in the future.

## 3. Methods

### 3.1. Hypothesis

The hypothesis of this study was as follows: In the absence of an emotional expression guide, work-related physical health problems, such as backache, headache, upper limb muscular pain, lower limb muscular pain, abdominal pain and general fatigue, as well as psychosocial health problems such as depressive symptoms, will be increased.

### 3.2. Study Population and Data Collection

Data were collected from the 4th Korean Working Condition Survey (KWCS) conducted by the Korea Occupational Safety and Health Agency (KOSHA) and developed based on the European Working Condition Survey. Data were collected after the Institutional Review Board at Y University granted permission (CR317347). After selecting eligible subjects for the survey, the researchers visited the subjects at their homes to collect survey data on their sociodemographic characteristics, working environment, work-related matters, relationships within the workplace, accidents and health care in the workplace. The post stratified estimation method was adopted to increase the national representation of the survey results.

**Figure 1:**
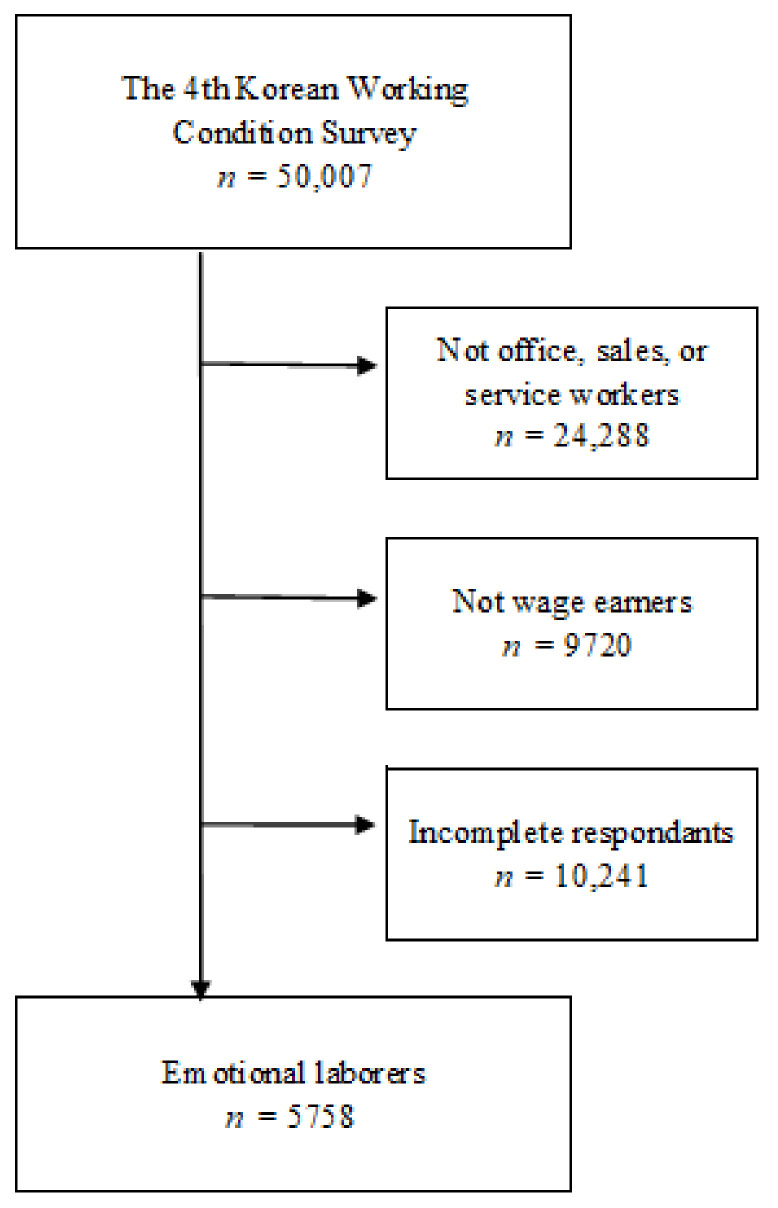
Flow chart of the study population selected for the analysis.

The process for selecting emotional workers was as follows. Considering the previous study and Hochschild’s definition, we selected the two survey questions to define emotional labor: “Does your job requires that you hide your feelings?” and “Do you deal directly with people who are not employees at your workplace, such as customers, passengers, pupils and patients?” To reduce heterogeneity and to focus on emotional labor, we excluded employers, blue collar workers, soldiers, administrative workers, and agriculture, forestry, and fishery workers. Sales workers, office workers and service workers were included in this study. Furthermore, we excluded laborers with missing values for variables of interest, including possible confounders. Finally, 5758 workers were included in the analysis (Figure 1).

### 3.3. Data Analysis

The data were analyzed by *t*-tests, χ^2^-test, correlation tests and univariate and multivariate logistic regression analyses using SPSS/WIN 22.0 (SPSS Inc., an IBM Company, Seoul, Korea) statistical software for Windows. We determined 95% confidence intervals (95% CIs) for each variable from the means and standard deviations. A *p*-value of <0.05 was deemed to indicate statistical significance. Weight estimates were also considered.

For the multivariate logistic analysis, we selected variables according to the significance of their odds ratio (OR) in the univariate logistic regression model and putative factors deemed to influence health problems. There was no multicollinearity, and the variance inflation factor was 1.042–1.295, not exceeding the criterion of 10.0. Moreover, to verify the model fit of the regression analysis, a Hosmer-Lemeshow test was conducted to confirm that the differences between the predicted and observed values were not significant (*p* = 0.357). In addition, the Akaike’s information criteria value was estimated to identify the fit of the final model. The results showed that the study’s final regression equation satisfied all of the assumptions for the regression equations, and thus the regression analysis results were deemed reliable.

### 3.4. Variable Measurements

#### 3.4.1. Independent Variable

An emotional expression guide was adopted as the independent variable in this study. The emotional expression guide was intended to protect emotional workers by establishing, in advance, the principles and standards of how to respond to the problems of customers. In general, companies provide emotional labor manuals in the form of booklets at work sites, so that they can find guidelines at any time when they have difficulty responding to customers.

The presence of an emotional expression guide was defined through questionnaires in this study. Workers who answered “yes” to the question “Is there an emotional expression guide manual (or norm) in relation to your job performance in your workplace?” were considered to have an emotional expression guide.

#### 3.4.2. Dependent Variables

This study considered health problems, including physical and psychosocial factors, as dependent variables. More specifically, backache, headache, muscular pain and abdominal pain were considered physical health problems. Depressive symptoms, overall fatigue, and sleep disturbance were included as psychosocial health problems. The presence of health problems was defined through questionnaires. Workers who answered “yes” to the question “Over the last 12 months, did you suffer from any of the following health problems?” were considered to have health problems. The presence of muscular pain was defined as having pains in the shoulders, neck, and/or upper and/or lower limbs such as hips, legs, knees and feet.

#### 3.4.3. Confounding Variables

##### Socio-Demographic Characteristics

Sociodemographic characteristics of sex, age, education and income were assessed. Education levels were divided into two categories (high school or less, and college or university), while monthly income levels were divided into quartiles (<100, 100–199, 200–299, and ≥300 thousand won) based on the mean value for the household income level of the study population.

##### Working Conditions

Data on the size of the workplace, weekly working hours and exposure to ergonomic risk factors were utilized to assess working conditions. The size of the workplace was divided into small business sites with less than 50 employees, medium-sized business sites with between 50 and 300 employees and large-scale business sites with more than 300 employees. Working hours per week were classified into either 40 or less hours or more than 40 h per week. The response of ergonomic risk factors and intensity of work was define as the ratio of the hours exposed during the working hours to the following six factors: all of the time, around three-quarters of the time, around half of the time, one quarter of the time, almost never, or never. One point was assigned for “exposure of more than half of working hours.”

## 4. Results

### 4.1. General Characteristics of the Study Population

A total of 5758 participants were included in our study, the data being sourced from the 4th KWCS. Of the participants, 63.6% were female and 36.4% were male workers, and a total of 57.8% of the study population were in their 30s or 40s. Approximately 56% of the subjects had an average monthly wage of less than 2 million won, and most of the emotional workers (79.5%) worked in small workplaces with less than 50 workers. Furthermore, of the total participant, 70.4% were highly exposed to ergonomic risk factors (Table 1).

### 4.2. Prevalence of Work-Related Health Problems According to the Existence of an Emotional Expression Guide

Table 2 presents the prevalence of work-related health problems according to the presence of an emotional expression guide. The work-related health problems of emotional labors were more prominent in the absence of an emotional expression guide. Among work-related health problems, lower limb muscular pain *(p* = 0.002), headache (*p* < 0.001) and general fatigue (*p* = 0.005) were considered to be more serious when there was no emotional expression guide. In addition, psychosocial health problems, such as depressive symptoms, were found to differ according to the existence of an emotional expression guide (*p* = 0.005).

### 4.3. Correlation between Emotional Expression Guide and Work-Related Health Problems

The absence of an expression guide negatively correlated with work-related health problems such as headache (*r* = −0.104, *p* = 0.001), lower limbs muscular pain (*r* = −0.080, *p* = 0.005), and general fatigue (*r* = −0.073, *p* = 0.009). Among job-related health problems, depressive symptoms were strongly negatively correlated with the absence of an emotional expression guide (*r*= −0.341, *p* = 0.006) (Table 3).

### 4.4. Odds Ratio of Work-Related Health Problems in the Absence of an Emotional Expression Guide

**Figure 2:**
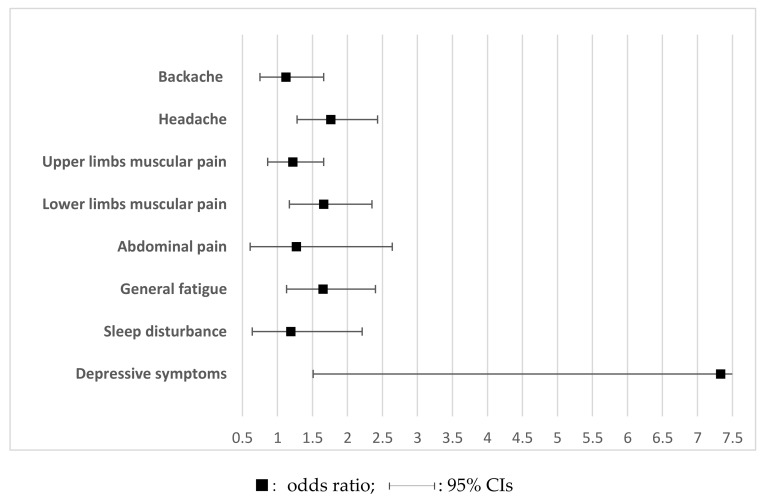
Associations between the emotional expression guide (reference: Yes) and work-related health problems in the univariate logistic regression.

Figure 2 presents the odds ratios and 95% CIs from univariate logistic regression analyses undertaken to identify work-related health problems that may arise if the emotional expression guide is not available. The results indicate that physical health problems, such as headaches, lower limb muscular pain and general fatigue, as well as psychosocial health problems such as depressive symptoms, were likely to occur in the absence of an emotional expression guide. Although not statistically significant, backache, upper limb muscular pain, abdominal pain and sleep disturbance were also indicated as work-related health problems in the absence of an emotional expression guide.

Table 4 showed how much job-related health problems increased in the absence of an emotion expression guide. More specifically, the odds ratio increased in headache (OR: 1.798; 95% CI: 1.288–2.508), lower limbs muscular pain (OR: 1.627; 95% CI: 1.130–2.342), general fatigue (OR: 1.582; 95% CI: 1.077–2.326) and depressive symptoms (OR: 6.149; 95% CI: 1.198–31.563) when there was no emotional expression guide. Although not statistically significant, OR also increased in headache, upper limb muscle pain, abdominal pain, and sleep disturbance.

## 5. Discussion

The results of this study showed that the proportion of female emotional workers was higher than that of male emotional workers because of management strategies that judge women to be more friendly to their customers than men [26]. According to Hochschild [1], emotional labor is a characteristic represented in jobs where kindness, sociality and femininity are emphasized. For this reason, previous studies asserted that women are a target group that requires emotional labor management [26,27]. Furthermore, this study showed that 42.4% of emotional workers earned a monthly salary of less than 2 million won. Considering that the average wage of Korean workers is 2970 thousand won [28], the wage level of emotional labor workers is low. Moreover, the statistics showed that the level of monthly wages of male workers was 1.5 times higher than that of female workers [28]. However, women were a more vulnerable group physically, psychologically and socioeconomically compared to men; therefore, both the health system and government policies should take additional steps to protect female emotional laborers.

Another risk factor that threatens the health of emotional laborers is the work environment, such as working in a small-sized workplace and/or being exposed to ergonomic risk factors. The results of this study showed that 79.5% of emotional laborers were working in small enterprises with less than 50 employees. Workers in small businesses are classified as a vulnerable group in term of health care management because small businesses with fewer than 50 employees are not required to appoint healthcare managers [29]. For this reason, workers working in small-sized workplaces have fewer opportunities for early disease screening and health-promotion education when compared to workers in medium to large businesses.

Furthermore, a previous study asserted that high emotional demands in the workplace have negative effects on health [6]. Accordingly, the government has established a health center for workers in small-sized workplaces to provide disease counseling, job stress management, work-related environment management, among other issues. Despite the government’s efforts, the number of work centers for workers in small-sized workplaces are still insufficient. Moreover, the problem is that health centers are not specifically for use by emotional laborers, so there are no health promotion programs or disease control programs exclusively for emotional laborers. Therefore, continuous efforts should be made, such as expanding and setting up health centers for workers, including emotional workers, to enhance the health equity of emotional laborers.

In general, emotional laborers are considered to have more psychological health problems such as stress, anxiety, depression and burn out than physical health problems. Previous studies on emotional labor concluded that emotional laborers are mainly salespersons, hotel workers and nurses. Looking at the characteristics of their work, they often stand all day long. Thus, emotional workers could easily be exposed to physical health problems, as well as psychological health problems. As the results of this study showed, 70.4% of the study population were exposed to ergonomic risk factors and were suffering from many physical health problems such as backache, upper or lower limb muscular pain, headaches and abdominal pain.

Many previous studies have argued that physical health problems are associated with psychosocial health problems because pain and mental health problems such as anxiety, depression and job-related stress share various biological pathways and neurotransmitters [16,30,31]. These studies have emphasized the need to confirm whether mental health problems are caused by physical health problems such as pain [30,32]. Emotional labor is known to have negative psychological consequences, such as emotional exhaustion, anxiety and depression [4,5]; therefore, the mental health problems of emotional workers could be exacerbated by physical health problems. Moreover, the health problems of emotional laborers need to be seriously considered because they could increase social costs by work interruption, lowered productivity and occupational disasters. As a result, increased organizational efforts and government management are required to protect emotional workers from health problems, such as by establishing policies, enacting related laws, and establishing facilities.

According to many previous studies on the effect of organizational support, there is a positive relationship between perceived organizational support and workers’ health [23,24,25]. Thanks to supportive organizations, employees may feel that the work stressor in question is less problematic, which may mitigate the contribution of the stressor to employee strain [21]. These positive effects have a great effect on the improvement of the physical and mental health of workers; therefore, organizational support that emotional workers can recognize should be further expanded. The Canadian government provided detailed guidelines to prevent health problems caused by emotional labor [31]. These guidelines play a role in managing and preventing negative factors that can arise from emotional labor. Workplaces protect the health of their emotional workers by developing guides tailored to the characteristics of the workplaces based on the guidelines in Canada [31]. However, in Korea, there is a lack of laws and regulations to protect emotional workers, and the employers’ interest in emotional workers’ health, as well as countermeasures to protect emotional laborers, are insufficient. A previous study asserted that in Eastern cultures that emphasize hierarchical order, it is difficult for workers to make legitimate demands for workers’ health protection [33]. In fact, workers do not even know that they can ask their employers to take measures to protect their health. Many studies have shown that in order to protect workers from personal health problems, researchers must consider various factors from the personal to the organizational level [15,16,17]. These studies have also confirmed that the health problems of emotional laborers could be reduced if organizations had emotional expression guides to protect their emotional workers. Therefore, organizations should not only take steps to protect emotional workers from health problems, but should also adopt an action-oriented system such as an emotional expression guide.

The limitations of this study were as follows. First, since this study used national data, the reliability of the study results was relatively high; however, the data used in this study were from 2014, and follow-up studies using the latest data are needed. Second, social support typically includes not only organizational support but also family, co-worker and friend support; however, in this study, social support at the interpersonal level was not considered. Third, this study was conducted in only one country. The response of workers to emotional guidance may differ depending on the culture. Therefore, the interpretation of this result could be limited. Finally, the presence or absence of disease may not be accurate because it was judged based on self-reported answers with a single question of the subjects, not based on doctors’ diagnoses. Therefore, intensive investigation of the relationship between organizational support and the work-related health problems of emotional workers should be conducted to elucidate the effect of an emotional expression guide on emotional workers’ health.

## 6. Conclusions

This study showed that emotional labor could be a major risk factor of the physical and psychosocial health problems of emotional workers. In this study, we demonstrated that an emotional expression guide is essential to prevent the mental and physical health problems of emotional workers. Therefore, attention and a national effort to protect the health of emotional workers should focus on the implementation of an emotional expression guide. In addition to the emotional expression guide, institutional mechanisms to protect emotional workers should be supported. Finally, we suggest a literature review to identify the effective means of guide construction for emotional workers in a follow-up study.

## Figures and Tables

**Table 1:** General characteristics of participants (*n* = 5758).

Characteristics	Classification	Total	Emotional Expression Guide		
			No	Yes	*p*-Value
Sex	Male	2094 (36.4)	1608 (76.8)	486 (23.2)	0.003
	Female	3664 (63.6)	2695 (76.3)	969 (26.4)	
Age (years)	≤29	1216 (21.1)	904 (74.3)	312 (25.7)	0.542
	30–49	3329 (57.8)	2475 (74.3)	854 (25.7)	
	50–69	1181 (20.5)	898 (76.0)	283 (24.0)	
	≥70	32 (0.6)	26 (81.3)	6 (18.8)	
Monthly Income (thousands of won)	<100	793 (13.8)	611 (77.0)	182 (23.0)	0.214
	100–199	2439 (42.4)	1830 (7.0)	609 (25.0)	
	200–299	1462 (25.4)	1068 (73.1)	394 (26.9)	
	≥300	1064 (18.5)	794 (74.6)	270 (25.4)	
Education	High school or less	2750 (47.8)	2061 (74.9)	689 (25.1)	0.377
	College or university	3008 (52.2)	2242 (74.5)	766 (25.5)	
Shift work	No	5122 (89.0)	3922 (76.6)	1200 (23.4)	<0.001
	Yes	636 (11.0)	381 (59.9)	255 (40.1)	
Number of workers	<50	4580 (79.5)	3500 (76.4)	1080 (23.6)	<0.001
	50–299	893 (15.5)	608 (68.1)	285 (31.9)	
	≥300	285 (4.9)	195 (68.4)	90 (31.6)	
Working time (hour/week)	≤40	3047 (52.9)	2255 (52.4)	792 (26.0)	0.101
	>40	2711 (47.1)	2048 (75.5)	663 (24.5)	
Exposed to ergonomic risk factor	Low	1703 (29.6)	1270 (74.6)	433 (25.4)	0.443
	High	4055 (70.4)	3033 (74.8)	1022 (25.2)	

**Table 2:** Prevalence of work-related health problems according to the existence of an emotional expression guide.

Classification	Emotional Expression Guide		*p*-Value
	No	Yes	
Backache	426 (68.7)	194 (31.3)	0.330
Upper limb muscular pain	1048 (72.2)	404 (27.8)	0.118
Lower limb muscular pain	710 (66.3)	361 (33.7)	0.002
Headache	573 (67.1)	281 (32.9)	<0.001
Abdominal pain	30 (63.8)	17 (36.2)	0.328
General fatigue	745 (67.1)	366 (32.9)	0.005
Sleep disturbance	60 (56.6)	46 (43.4)	0.344
Depressive symptoms	24 (60.0)	16 (40.0)	0.005

**Table 3:** Correlations among variables used in this study.

Variables	1	2	3	4	5	6	7	8	9	10	11	12	13	14	15
1.Emotional expression guide manual	1														
2. Age	−0.014	1													
3. Monthly Income	0.027 *	0.165 **	1												
4. Shift work	0.012 **	−0.084 **	0.140 **	1											
5. Number of workers	0.076 **	0.082 **	0.262 **	0.01	1										
6. Working time	−0.018	−0.012	0.084 **	0.015	−0.123 **	1									
7. Exposed to ergonomic risk factor	−0.002	0.138 **	0.256 **	0.114 **	−0.108 **	0.138 **	1								
8. Backache	−0.02	0.109 *	0.114	0.107 *	−0.038	0.207 **	0.233 **	1							
9. Headache	−0.104 **	0.120 **	0.266 **	0.021	−0.118 **	0.061 *	0.009	0.381 **	1						
10. Upper limbs muscular pain	−0.03	0.06	0.111 *	0.054 *	−0.046	0.126 **	0.234 **	0.746 **	0.443 **	1					
11. Lower limbs muscular pain	−0.08	0.113 **	0.117	0.045	−0.029	0.071 *	0.270 **	0.638 **	0.481 **	0.740 **	1				
12. Abdominal Pain	−0.052	0.21	0.284	0.036	−0.099	0.104	0.182 *	0.532 **	0.521 **	0.344 **	0.411 **	1			
13. General fatigue	−0.073 **	0.051 *	0.167 **	0.060 **	−0.040 *	0.092 **	0.339 **	0.171 **	0.027	0.165 **	0.137 **	0.019	1		
14. Sleep disturbance	−0.042	0.128	0.294	0.049	−0.081	0.170 *	0.054	0.486 **	0.497 **	0.468 **	0.426 **	0.544 **	0	1	
15. Depressive symptom	−0.341 **	0.274	0.458	0.128	−0.159	0.081	0.104	0.548 **	0.431 **	0.442 **	0.529 **	0.690 *	0	0.866 **	1

* *p* < 0.05, 2 tailed, ** *p* < 0.01, 2 tailed.

**Table 4:** Associations between the emotional expression guide (Reference: Yes) and work-related health problems in multivariate logistic regression.

Variables			Backache	Headache	Upper Limbs Muscular Pain	Lower Limbs Muscular Pain	Abdominal Pain	General Fatigue	Sleep Disturbance	Depressive Symptoms
			OR (95%CI, *p*)							
Model I	Emotional expression guide	Yes	1	1	1	1	1	1	1	1
		No	1.117 (0.751–1.661, 0.584)	1.762 (1.277–2.431, <0.001)	1.220 (0.859–1.663, 0.204)	1.655 (1.165–2.349, 0.004)	1.266 (0.607–2.638, 0.531)	1.645 (1.131–2.393, 0.007)	1.193 (0.643–2.211, 0.576)	7.333 (1.511–35.593, 0.004)
Model II	Emotional expression guide	Yes	1	1	1	1	1	1	1	1
		No	1.132 (0.760–1.685, 0.543)	1.806 (1.300–2.510, <0.001)	1.227 (0.899–1.675, 0.197)	1.655 (1.162–2.358, 0.005)	1.274 (0.595–2.731, 0.533)	1.636 (1.122–2.385, 0.011)	1.144 (0.603–2.170, 0.680)	6.927 (1.389–34.550, 0.018)
Model III	Emotional expression guide	Yes	1	1	1	1	1	1	1	1
		No	1.026 (0.672–1.568, 0.904)	1.798 (1.288–2.508, <0.001)	1.279 (0.924–1.769, 0.138)	1.627 (1.130–2.342, 0.009)	1.208 (0.540–2.703, 0.645)	1.582 (1.077–2.326, 0.020)	1.111 (0.575–2.148, 0.754)	6.149 (1.198–31.563, 0.030)

Model I: not adjusted. Model II: adjusted for personal factors (gender, age, education, monthly income). Model III: adjusted for Model II + working conditions (shift work, number of workers, working time, exposed to ergonomic risk factor).

## Data Availability

All data files are available from the Korea Occupational Safety and Health Agency database through the following URLs: https://kosha.or.kr, accessed on 19 June 2021. Anybody, including an international researcher who signs up for membership, can get raw data from the webpage.
